# Modeling the Role of Lanthionine Synthetase C-Like 2 (LANCL2) in the Modulation of Immune Responses to *Helicobacter pylori* Infection

**DOI:** 10.1371/journal.pone.0167440

**Published:** 2016-12-09

**Authors:** Andrew Leber, Josep Bassaganya-Riera, Nuria Tubau-Juni, Victoria Zoccoli-Rodriguez, Monica Viladomiu, Vida Abedi, Pinyi Lu, Raquel Hontecillas

**Affiliations:** Nutritional Immunology and Molecular Medicine Laboratory, Biocomplexity Institute of Virginia Tech, Blacksburg, Virginia, United States of America; Vanderbilt University Medical Center, UNITED STATES

## Abstract

Immune responses to *Helicobacter pylori* are orchestrated through complex balances of host-bacterial interactions, including inflammatory and regulatory immune responses across scales that can lead to the development of the gastric disease or the promotion of beneficial systemic effects. While inflammation in response to the bacterium has been reasonably characterized, the regulatory pathways that contribute to preventing inflammatory events during *H*. *pylori* infection are incompletely understood. To aid in this effort, we have generated a computational model incorporating recent developments in the understanding of *H*. *pylori*-host interactions. Sensitivity analysis of this model reveals that a regulatory macrophage population is critical in maintaining high *H*. *pylori* colonization without the generation of an inflammatory response. To address how this myeloid cell subset arises, we developed a second model describing an intracellular signaling network for the differentiation of macrophages. Modeling studies predicted that LANCL2 is a central regulator of inflammatory and effector pathways and its activation promotes regulatory responses characterized by IL-10 production while suppressing effector responses. The predicted impairment of regulatory macrophage differentiation by the loss of LANCL2 was simulated based on multiscale linkages between the tissue-level gastric mucosa and the intracellular models. The simulated deletion of LANCL2 resulted in a greater clearance of *H*. *pylori*, but also greater IFNγ responses and damage to the epithelium. The model predictions were validated within a mouse model of *H*. *pylori* colonization in wild-type (WT), LANCL2 whole body KO and myeloid-specific LANCL2-/- (LANCL2^Myeloid^) mice, which displayed similar decreases in *H*. *pylori* burden, CX3CR1+ IL-10-producing macrophages, and type 1 regulatory (Tr1) T cells. This study shows the importance of LANCL2 in the induction of regulatory responses in macrophages and T cells during *H*. *pylori* infection.

## Introduction

*H*. *pylori* is the dominant indigenous member of the human gastric microbiota present in roughly half of the world’s population [1]. Since the isolation of *H*. *pylori* from patients with peptic ulceration, the association between this microaerophilic bacterium and gastric diseases has grown stronger [2,3,4,5]. However, *H*. *pylori* has co-evolved with humans for thousands of years, and the majority of *H*. *pylori*-colonized individuals, an estimated 85%, do not present any detrimental effects [6]. Growing, and sometimes contradictory evidence, suggests these individuals may be deriving benefits from *H*. *pylori* against a broad range of disorders, including asthma and metabolic diseases [7,8,9]. Therefore, the events that promote tolerance to the bacterium in the gastrointestinal mucosa and systemic regulatory effects merit further investigation.

Immune responses resulting from IFNγ and IL-21-producing T cells have been shown to be responsible for *H*. *pylori*-associated gastric inflammation [10,11,12]. Additionally, dendritic cells (DC) have been thought to be the dominant mechanism for sensing *H*. *pylori* in the gastric environment [13,14]. However, other myeloid cell subsets may contribute to shaping the balance between effector and regulatory responses. For instance, we hypothesize that a population of CX3CR1+ macrophages may be required for the promotion of a regulatory environment that balances excessive inflammation and effector responses. In support of this hypothesis, this population has been associated with beneficial effects in inflammatory bowel disease (IBD), allergic responses in the lung, gastrointestinal cancers and steatohepatitis [15,16,17,18]. As a cell type that has only recently gained prominence, relatively little is known about the differentiation into and maturation of this phenotype. An important aspect of the functional characterization of CX3CR1+ regulatory macrophages is that it produces IL-10, a major regulatory cytokine [19]. The production of IL-10 is self-amplifying as the binding of IL-10 to the IL-10-receptor in myeloid cells activates STAT3, which controls downstream transcriptional activities [20]. In general, the maturation and stimulation of macrophages are controlled by the cytokine M-CSF and its associated receptor CSF1R [21]. However, the stimulation of CSF1R is associated with NFκB pathways that also associate with IFNγ and IL1β inflammatory responses [22,23]. Finally, one of the key phenotypic markers is CX3CR1, the fractalkine receptor, capable of producing a variety of downstream effects on secondary messenger and PI3K pathways [24]. Expression of the receptor is regulated in part by members of the Kruppel-like factor family [25].

We applied computational modeling approaches for generating a systems-wide view of the massively and dynamically interacting complex immune responses to *H*. *pylori* [26]. Prior efforts to model *H*. *pylori*-host interactions have been successful in investigating the roles of various T cell subsets particularly the balance between T helper 17, Th17, and induced T regulatory, iTreg, subsets and between T follicular helper and T follicular regulatory cells [27,28]. Similar models have generated hidden informative insights into the immune system’s role in IBD and *C*. *difficile* infection [29,30]. The successes of computational approaches in modeling mucosal immune responses also extend to a finer scale of resolution with the ability to assess intracellular mechanisms controlling differentiation of cell types including the role of NLRX1 in the differentiation of inflammatory macrophages in response to *H*. *pylori* [31,32]. Additionally, we have developed a sensitivity analysis method for agent based modeling that has indicated importance of a regulatory macrophage cell type in response to *H*. *pylori* [33].

This study has generated two new computational models to assess a wide array of cellular and molecular events implicated in immune response to *H*. *pylori* colonization. The first model aims to address which cell type is most critical in promoting a regulatory response at the gastric mucosa. The model encompasses a multi-compartment view of the gastric mucosa, including immune cells, epithelial cells, and bacteria. The second model aims to address what intracellular pathways drive the differentiation of regulatory macrophages and it is based on an intracellular signaling network. From this network, we determine that LANCL2 signaling is required for sufficient differentiation of regulatory macrophages to allow for maintenance of a dominantly regulatory gastric environment. The alteration of regulatory macrophage differentiation, intracellularly, is predicted to diminish IL10 production resulting in lower bacterial loads and increased late-phase effector responses mediated by IFNγ in the tissue-level model. The conclusions of this modeling effort are validated experimentally with a mouse model of *H*. *pylori* infection.

## Results

### Tissue level computational model of immune responses to *H*. *pylori* infection

**Fig 1:**
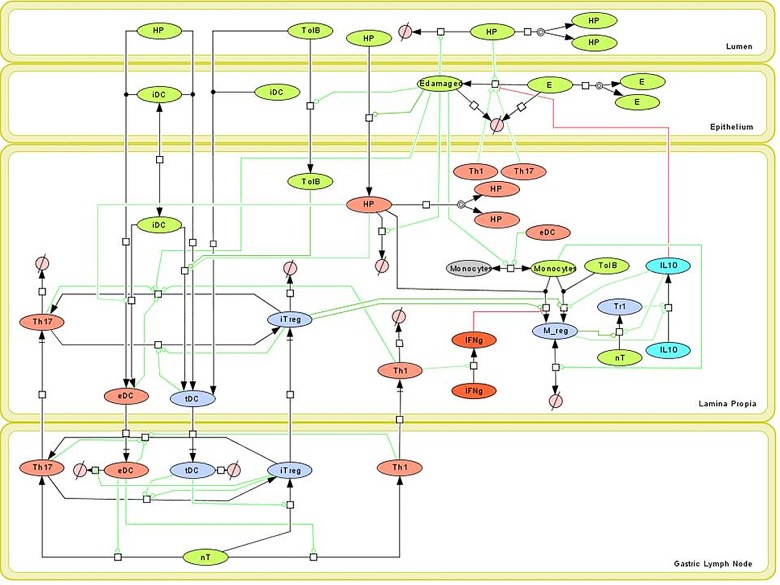
Network model of *Helicobacter pylori*-host interactions. Systems biology markup language (SBML)-compliant network diagram of four compartment *H*. *pylori* response model. Cell types include *H*. *pylori* (HP), tolerogenic bacteria (TolB), epithelial cells (E), damaged epithelial cells (Edamaged), immature dendritic cells (iDC), effector dendritic cells (eDC), tolerogenic dendritic cells (tDC), monocytes (Monocytes), regulatory macrophages (M_reg), naïve T cells (nT), T helper 1 (Th1), T helper 17 (Th17), induced T regulatory (iTreg), type 1 regulatory (Tr1), IFNγ (IFNg), and IL10 (IL10).

Based on new knowledge generated by the interactions of *H*. *pylori* with the gastric mucosa, we constructed a network model describing the immune responses resulting from *H*. *pylori* infection (Fig 1) [27]. In particular, the refined model revises the regulatory and IL-10-driven responses initiated by the interaction of *H*. *pylori* with myeloid cells. The model includes four compartments. The lumen contains *H*. *pylori* (HP) and other commensal bacteria (TolB). The epithelium contains epithelial cells existing in two states healthy (E) and damaged (Edamaged) as well as infiltrating immature dendritic cells (iDC). The lamina propria (LP) contains infiltrating TolB and HP, cytokines IL-10 and IFNy, and a variety of immune cells: immature dendritic cells (iDC), effector dendritic cells (eDC), tolerogenic dendritic cells (tDC), monocytes (Monocytes), regulatory macrophages (Mreg), T helper 1 (Th1), T helper 17 (Th17), induced T regulatory (iTreg), type 1 regulatory (Tr1) and naïve CD4+ T cells (nT). The gastric lymph node compartment contains eDC, tDC, Th1, Th17, iTreg, and nT. With the updated network in place, ordinary differential equations (ODE) were assigned to each species within the model (Fig 1). Equations encompassed both simple and Hill-type activation and inhibition and are provided in full within S1 File. In total, the model contains 36 ordinary differential equations (ODEs) containing 77 parameters provided within S1 File.

### Model calibration with experimental data and analysis using local and global sensitivity analyses

**Fig 2:**
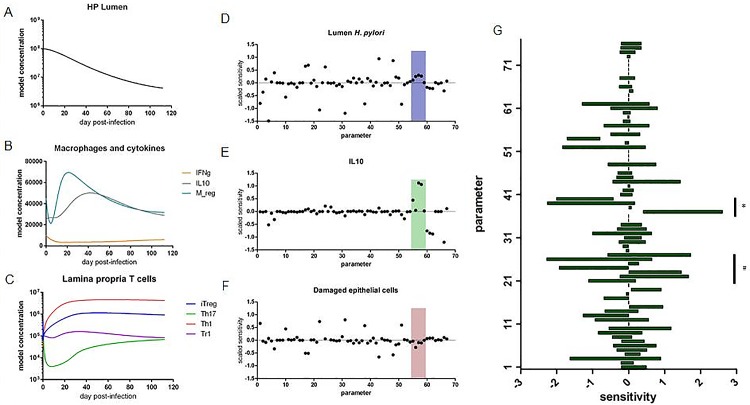
Simulation and analysis of *Helicobacter pylori* colonization. Time course simulation with calibrated *H*. *pylori*-host interaction model displaying *H*. *pylori* burden (A), macrophage and cytokine responses (B) and T cell responses (C). Local sensitivity analysis of model displaying positive and negative effects of parameters on model species H. pylori (D), IL10 (E), damaged epithelial cells (F). Boxed region contains parameters associated with Mreg model species. Specific model species of interest are indicated by symbols: & marks column associated with HP model species, * marks column associated with IL10 model species and # marks column associated with Edamaged model species. Global sensitivity analysis displaying parameter sensitivity ranges on the differentiation of regulatory macrophages (G). Parameters associated with cytokines are indicated by (*) and parameters associated with *H*. *pylori* are indicated by (#).

We obtained data from a mouse model of *H*. *pylori* infection to calibrate the model. Briefly, stomachs were collected at various time points post infection for up to 16 weeks. Portions of the stomach were dedicated to the analysis of *H*. *pylori* burden through bacterial culture and immune cell phenotyping by flow cytometry. With this data, we then calibrated the model on a global scale. Data provided within S2 File. All parameter values within the model were allowed to vary to fit the data. Using the global search optimization method, Particle Swarm, the model was calibrated to recapitulate the immune, and bacterial dynamics observed over the time course of infection (Fig 2A–2C). The calibrated model was assessed by local and global sensitivity analyses. In the local sensitivity analysis (SA), the effect of each parameter, near its calibrated parameter value, on each of the model species was calculated. The local SA was performed in COPASI. This enabled the evaluation of the most impactful interactions affecting model species of interest, such as HP, IL10 or Edamaged (Fig 2D–2F). Interestingly, parameters related to the Mreg species (boxed region) were identified as having a large effect on decreasing the Edamaged species while maintaining the HP population (Fig 2D and 2F). The five Mreg parameters defined (from left to right): 1) proliferation/recruitment of precursor cells, 2) *H*. *pylori* recognition, 3) activation of regulatory differentiation, 4) activation of macrophage IL-10 production and 5) death rate of macrophages. In particular, the proliferation/recruitment, differentiation and cytokine production parameters appear to be the most impactful on the species of interest. Following this insight, we focused on the Mreg population for global sensitivity analysis. Global sensitivity analyses, performed in Condor COPASI, allow for the determination of a range of sensitivity for each parameter based on assessing the sensitivity throughout the entire allowable parameter space [34]. The Mreg species was identified to be highly sensitive to both the cytokine environment and the overall level of *H*. *pylori* present (Fig 2G).

### Computational model of intracellular pathways in regulatory macrophages

**Fig 3:**
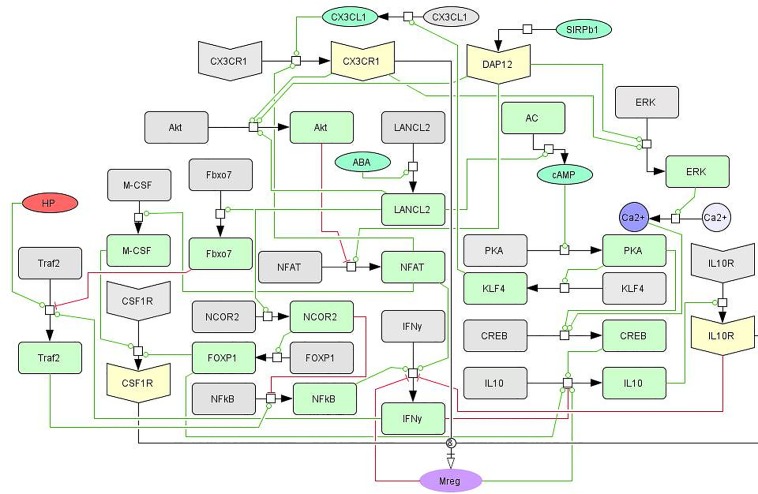
Network of intracellular signaling pathways controlling regulatory macrophage differentiation. Systems Biologt Markup Language (SBML)-compliant network diagram displaying the three main pathways, CSF1R, CX3CR1, and IL-10R, controlling cytokine production and macrophage phenotype commitment. While interconnected, the three signaling receptors have dominant effects on arms within the network. The CSF1R/M-CSF pathway integrates DAP12 and NFAT activity and is closely associated with the inflammatory Traf/NF-kB pathway as well. The IL-10R pathway is modified by calcium signaling and the transcriptional activities of FOXP1 and NCOR2. The CX3CR1 pathway combines cAMP/PKA pathways with modification to Akt and inflammatory signaling.

Following the identification of regulatory macrophages, via SA, as crucial controllers of gastric inflammation in response to *H*. *pylori*, an intracellular model of macrophage differentiation was developed (Fig 3). The model determines the ability of a macrophage to differentiate into a regulatory phenotype based on the signaling pathways from three receptors: IL10R, CX3CR1, and CSF1R. The network model was first constructed through a comprehensive literature search on the differentiation of regulatory and tissue-resident macrophages. It was further enhanced by pathway analysis of genes and signaling mechanisms associated with the initial network topology. The network contains phosphorylation events, receptor-ligand interactions, transcriptional regulation, generation and release of secondary messengers and cytokine production portrayed through state transitions, activations, and inhibitions. Importantly, the intracellular model contains linkages to the cellular tissue level model through HP, IFNg, and IL-10 species. The resultant differential equations were assigned mass action or Hill-type dynamics. The model is comprised of 40 differential equations containing 132 parameters. The ODEs were calibrated using a combination of sourced and new data generated from *in vitro* macrophage differentiation studies, compiled into a dataset provided within S2 File.

### SA of intracellular pathways in Mreg and validation of modeling predictions

**Fig 4:**
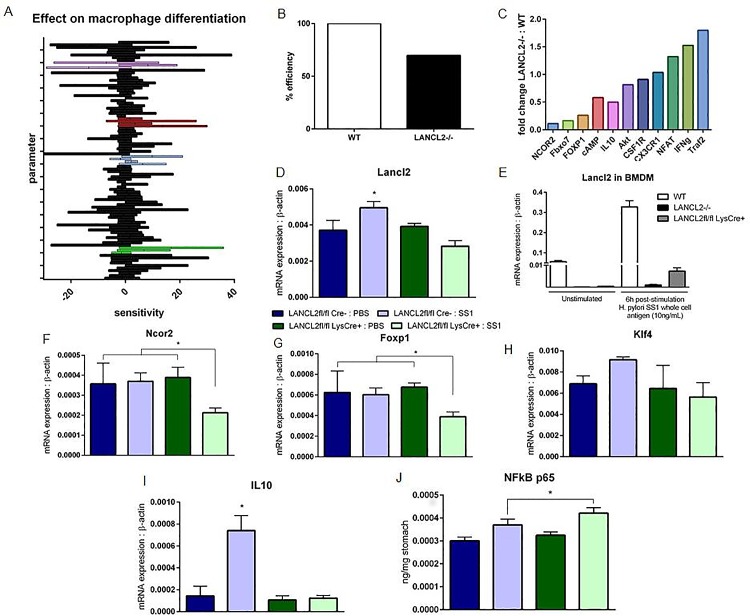
Simulation, analysis and validation of intracellular macrophage network model. Global sensitivity analysis displaying parameter sensitivity ranges on the differentiation of regulatory macrophages (A). Parameters are grouped by associated model species: NFkB-associated (purple), LANCL2-associated (red), PKA-associated (blue), IL10-associated (green). Predicted impairment of regulatory macrophage differentiation following simulated loss of LANCL2 (B). Fold changes in molecule production or activation following simulated loss of LANCL2 compared to wild-type simulation (C). mRNA expression of LANCL2 in whole stomach (D) and cultured bone marrow derived macrophages (E), normalized to expression of beta-actin. mRNA expression of Ncor2 (F) and Foxp1 (G), Klf4 (H), and Il10 (I) within the stomach of *H*. *pylori* SS1-infected and uninfected LANCL2fl/fl; LysCre- and LANCL2fl/fl LysCre+ mice at three weeks post-infection. NFkB p65 activity in whole stomach tissue homogenate of *H*. *pylori* SS1-infected and uninfected LANCL2fl/fl; LysCre- and LANCL2fl/fl LysCre+ mice at three weeks post-infection (J). *p*-values less than 0.05 are considered significant and marked by an asterisk (*), (n = 7).

Global SA was performed on the calibrated model to assess the effects of all parameters on the Mreg species (Fig 4A). A few of the largest sensitivities were expected results, such as a large positive effect by IL-10 signaling and a largely negative effect by NF-κB signaling. Additionally, the CSF1R pathway was highlighted as a critical element in the network through showing mixed effects suggesting that a balance is needed in CSF1R driven responses allowing the activation of a macrophage without overloading the signal driving the macrophage to an inflammatory state. Aside from established mechanisms previously explored, one node within the network also showed comparable positive effects to IL-10 signaling: LANCL2 (Fig 4A). Through reduction of initial inactivated and activated LANCL2 to zero, an *in silico* knockout of LANCL2 was generated. When simulated with LANCL2 knocked out, the ability of a macrophage to differentiate into a regulatory phenotype is decreased to 70% of the simulated wild-type model (Fig 4B). Additionally, large changes are observed throughout the model on other nodes of the network after knocking out LANCL2 (Fig 4C). Transcriptional regulators NCOR2, Fbxo7 and FOXP1 were greatly decreased in activity. Meanwhile, inflammatory pathways, Traf2, IFNg, and NFAT, were increased following simulated LANCL2 knockout. The effect of LANCL2 on other regulatory nodes within the network was validated with a mouse model of *H*. *pylori* infection. Real-time RT-PCR was conducted on RNA isolated from sections of the stomach from infected and non-infected mice. LANCL2fl/fl LysCre- (a wild-type phenotype) and LANCL2fl/fl LysCre+ (a myeloid specific knockout of LANCL2) groups were compared in the analysis (S4 File). *H*. *pylori* SS1 infected LANCL2fl/fl LysCre- mice displayed higher expression of LANCL2 than uninfected control, a response that was absent in LANCL2fl/fl LysCre+ mice (Fig 4E). Mice lacking LANCL2 in myeloid cells showed significantly lower expression of Ncor2 and Foxp1 when infected with *H*. *pylori* (Fig 4F and 4G) while Klf4 expression was increased only within LANCL2fl/fl LysCre- infected mice. IL-10 expression was also significantly increased only within LANCL2fl/fl LysCre- infected mice (Fig 4I). In contrast, LANCL2fl/fl LysCre+ mice infected with *H*. *pylori* had an increased activity of NFkB p65 within the whole stomach (Fig 4J).

### Multiscale modeling of the role of LANCL2 at the tissue level

**Fig 5:**
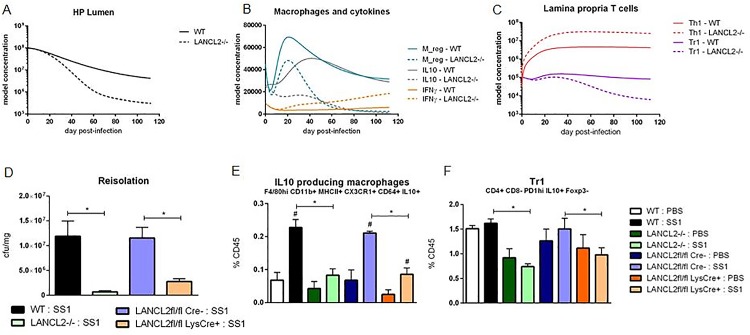
Simulation and validation of the effects of LANCL2 loss on the response to *Helicobacter pylori* colonization. Simulated time course effects of LANCL2 knockout on *H*. *pylori* burden (A), macrophages and cytokines (B), and lamina propria T cells (C). Bacterial re-isolation of *H*. *pylori* from stomachs of wild-type (black), LANCL2-/- (green), LANCL2fl/fl; LysCre- (blue) and LANCL2fl/fl; LysCre+ (orange) mice at week three post-infection (D). Percentage of IL10+ CX3CR1+ CD64+ MHCII+ CD11b+ F4/80^hi^ macrophages (E) and IL10+ FOXP3- PD1^hi^ type 1 regulatory T cells (F) at week 3 post-infection in wild-type, LANCL2-/-, LANCL2fl/fl; LysCre-, and LANCL2fl/fl; LysCre+ mice. p-values less than 0.05 are considered significant and marked by an asterisk (*) for genotype differences and a pound sign (#) for treatment differences within genotype, (n = 7).

The predicted efficiency of Mreg differentiation following LANCL2 knockout was then used to simulate the cellular tissue level model of the gastric mucosa during *H*. *pylori* infection. Through an adjustment of Mreg parameters to provide 70% of the Mreg population at its peak, an *in silico* macrophage deletion of LANCL2 was created. Following this deletion, a large decrease in the *H*. *pylori* burden was observed (Fig 5A). A shift from an IL-10 dominated cytokine environment to an IFNγ dominated cytokine environment occurred simultaneously with the initiation of *H*. *pylori* burden differences (Fig 5B). Further, the T cell environment shifted noticeably towards a Th1 and Th17 centric effector CD4+ T cell responses as opposed to the balanced responses observed in the wild type simulation (Fig 5C). After observing the broad effects of LANCL2 knockout *in silico*, we validated the predictions in our mouse model of *H*. *pylori* infection with LANCL2*-/-* and myeloid cell-specific LANCL2^Myeloid^ (LANCL2fl/fl; LysCre+) mice. The mouse model of infection included the same strain and dosing strategy as the model used to generate wild-type calibration data. As predicted, the gastric luminal burden of *H*. *pylori* was significantly decreased in mice lacking LANCL2 in all cells or only myeloid cells (Fig 5D). A significant reduction in CX3CR1+ IL10-producing macrophages, the *in vivo* analog of the Mreg species, at three weeks post infection was observed, resulting in a decrease to 50% of wild-type levels in LANCL2-/- mice (Fig 5E). The myeloid-specific deletion of LANCL2 recapitulated this reduction in regulatory macrophages. A reduction in Tr1 (CD4+FOXP3-PD1^hi^IL10+) cells was also observed at three weeks post infection, closely mirroring the predicted 50% reduction at that time (Fig 5F). While slightly higher levels of Tr1 cells exist in the LANCL2fl/fl LysCre+ mice, a significant reduction compared to the LANCL2fl/fl LysCre- mice is still present, suggesting a downstream effect of the regulatory macrophage population on the differentiation of T cells.

## Discussion

*H*. *pylori* is the dominant indigenous bacterium of the gastric microbiota. The microbe can colonize the stomach without the generation of adverse effects, with little to no activation of inflammatory pathways, in the majority of individuals. However, with age, certain members of the population lose tolerance to the bacterium contributing to the development of chronic gastric diseases. Computational modeling can be used to simulate "what if" conditions and test the effect of perturbations, genetic modifications, or simply aid in understanding how complex biological systems change over time [35]. By using simulations to study behavior of systems and the impact of perturbations, one can investigate dependencies, systems robustness and fragility, and resilience, from molecular levels to tissue levels [36]. In this work, we apply modeling in combination with immunology experimentation to identify and validate important contributors to the retention of tolerance through the combination of computational and experimental methods.

The update and analysis of the tissue level model of the gastric mucosa, including lumen, epithelium, LP and gastric lymph nodes, confirmed recent experimental developments on the importance of regulatory macrophages on the immune response to *H*. *pylori* infection [10,27]. These regulatory macrophage populations are predicted to favor regulatory environment that increase *H*. *pylori* colonization and prevent epithelial cell damage. Both *in vivo* and *in silico*, these actions are likely the result of the macrophage-derived production of IL-10 in the gut mucosa. The cytokine has broad immunoregulatory effects in the promotion of tolerogenic cell types, such as iTreg and Tr1 cells, and the maintenance of epithelial cell health [37]. While *H*. *pylori* colonizes the gastric mucosa, these macrophage populations might suppress the inflammatory response that can lead to the development of ulcers or gastric cancers.

Indeed, modeling and experimental work has focused on the development of CD4+ T cell populations in response to *H*. *pylori* [10,27,31]. However, both recent experimental evidence and the computational predictions of the updated model suggest that these CD4+ T cell events are downstream effectors under the control of a regulatory macrophage population. Most notably, Tr1 cells, a regulatory subset whose expansion is largely dependent on environmental IL-10, has a distinct and short-lived peak coinciding with the expansion of regulatory macrophages [38]. IL-10 signaling is also important in establishing the immunoregulatory effects of Treg cells through STAT3 phosphorylation and the self-amplifying increase in IL-10 production [39,40]. Within effector T cells, IL-10 has been shown to decrease the expression of RORγT in Th17 cells and general production of inflammatory cytokines [41]. The second crucial event occurs around day 25, when the majority of the CD4+ T cell populations begin to increase in the gastric mucosa. While the regulatory macrophage population has begun to decrease by this point, noticeable differences between wild-type and LANCL2-/- exist, both computationally and experimentally. Prior to this point, the only noticeable difference is a reduction in regulatory macrophages; but after this point, the *H*. *pylori* burden begins to diverge indicating an effector response involving CD4+ T cells. This suggests that the macrophage population is critical in establishing a lasting homeostatic response through the promoted regulatory environment during initial exposure events.

As these regulatory macrophages are of high functional importance during the immune response to *H*. *pylori* and in the development of other gastrointestinal diseases such as IBD, we developed an intracellular network model describing the differentiation and function of macrophages into this regulatory phenotype. The model, comprised of known and theoretical connections to macrophage differentiation, is an important step for the understanding of the CX3CR1+ macrophage population. Sparse knowledge exists on the development of Mreg, but the population has recently been implicated in IBD, cancer, and liver disease in addition to *H*. *pylori* infection [15,17,42,43]. Computational modeling can greatly accelerate the evaluation of potential mechanisms influencing the establishment of regulatory CX3CR1+ macrophage populations and their linkages to lesion formation. In particular, the assembly of a network, featuring over 20 nodes, identified LANCL2 as a core component of macrophage differentiation. This key molecular controller was validated to be important in the establishment of regulatory responses to *H*. *pylori* and the continued colonization of the gastric environment by the microbe.

Importantly, pathways associated with the activation of macrophages are often closely associated with inflammatory responses, such as through the cytokine, M-CSF [44,45,46]. Therefore, a balance in activation signals is needed that attenuates signaling once an initial stimulus threshold is reached based on inflammatory and metabolic environmental cues. To induce a regulatory macrophage subset, however, the production of IL-10 must also be stimulated. Based on the development and refinement of the model network, we predict the transcriptional activities of FOXP1 and CREB to be critical in the establishment of the IL-10 response in regulatory macrophages.

Modeling studies identified LANCL2, a membrane receptor previously associated with effects on Akt and glucose regulation pathways [47,48,49], as a key factor in controlling Mreg. Recently, activation of LANCL2 has been connected with the promotion of immunoregulatory effects within multiple cell types including epithelial cells and T cells [47]. Indeed, LANCL2-/- mice showed even greater reduction in *H*. *pylori* burden at three weeks post infection compared to the LANCL2^Myeloid^ KO strain, suggesting additional LANCL2 epithelial and/or T cell intrinsic contributions to the regulatory environment of the mucosa. The membrane receptor has one known natural endogeneous ligand, abscisic acid, but additional synthetic compounds have been discovered to promote similar pharmacological effects [48,50]. The predicted impact of LANCL2 suggests that many of these effects result from an influence on cellular differentiation, particularly on the differentiation of CX3CR1+ macrophages. Through the simulated loss of LANCL2, we show decreases in the activation of transcriptional regulators and the production of secondary messengers predicted to be partly responsible for driving IL-10 production. Among the transcription factor changes, the most notable were with FOXP1 and NCOR2, previously shown to work cooperatively in the repression of c-fms, the gene encoding the M-CSF receptor, CSF1R [51]. Also greatly downregulated was the ubiquitin ligase, Fbxo7. This protein is likely essential for LANCL2 mediated downregulation of inflammatory pathways because of its previously explored abilities to mark important TNF and TRAF signaling molecules for degradation [52]. Notably, Fbxo7 and members of the LanC-like family have been shown to impact similar neurological disorders [53,54]. Moreover, LANCL2 is important for the production of secondary messengers such as cAMP and Ca^2+^, which help to drive PKA and calmodulin events needed for the elevated production of IL-10 [48].

While the presented models were able to identify important cell types and signaling molecules implicated in the immunoregulatory mechanisms to *H*. *pylori* infection, a number of simplifications and weaknesses could be improved upon in future modeling work. Firstly, while the model is well-calibrated and representative of cell responses at the population level, direct cell-to-cell interactions are not apparent with the current modeling method. The model was able to identify that regulatory macrophages are important and promoting effects through IL-10 production. However, questions of how the macrophage interacts with *H*. *pylori*, or what the migratory behavior of the macrophage is within the compartment cannot be addressed. To improve this and the overall granularity of the model, the presented model could be used as a scaffold to generate an agent based model (ABM) of the system to allow individual cell decisions and changes in the spatial microenvironment [55,56]. Secondly, the model system described provides great depth into the behavior of macrophages but is more superficial on the other cell types of the model. While driven by the sensitivity analysis suggesting macrophages would be the most important cell to develop in detail, the predictive capacity of the model may be further improved by incorporating similar cell-specific models, for T cells, dendritic cells or epithelial cells, to inform the behavior of all cells included in the tissue level model.

Validating the computational hypotheses of the presented models, the loss of LANCL2 in mice, both throughout the full body and only within myeloid cells, results in a decreased burden of *H*. *pylori* within the gastric mucosa. In WT, a dramatic increase in IL-10 producing cells is observed at three weeks post-infection. The IL-10 driven regulatory responses suppress the generation of inflammatory responses by T cells and the recruitment of cells responsible for bacterial clearance. Through the loss of this response in LANCL2-/- and LANCL2^Myeloid^ mice, the *H*. *pylori* burden is greatly reduced beginning around week three post-challenge. However, the change in bacterial burden is also accompanied by significant decreases in immunoregulatory cell types in the gastric LP. While the bacterial burden is lower in these mice, the continued exposure to *H*. *pylori* combined with the decrease in immunoregulation is likely to result in the development of symptomatic disease similar to the pathologies developed in roughly 15% of the world’s *H*. *pylori*-colonized population. With decreases in IL-10, epithelial cells are more likely to have higher turnover rates, which in parallel low-grade inflammation can have slight mutagenic effects [57,58,59,60]. Therefore, the stimulation of CX3CR1+ macrophages is an important consideration in the treatment of *H*. *pylori*-associated health issues. Potentially, the use of LANCL2 ligands could ameliorate clinical signs of disease in *H*. *pylori* colonized individuals and serve as an alternative to antibiotic administration.

Computational modeling of the immunological changes within the gastric mucosa following *H*. *pylori* infection predicted the importance of regulatory macrophages in mediating the immunoregulatory mechanisms during *H*. *pylori* infection. Regulatory macrophages promote tolerance to the bacterium while maintaining epithelial integrity by producing IL-10. Changes in IL-10 concentration in the gastric environment additionally alter T cell differentiation, increasing the presence of Tr1 cells, which further aid in the maintenance of immunological homeostasis. With the absence or reduction of these regulatory pathways early in infection, late stage increases in IFNγ occur serving as a marker of inflammation. The development of an intracellular model predicted the importance of balancing activation and regulatory signals during the differentiation process, for which LANCL2 was critical. In particular LANCL2 was predicted to alter the activity of IL-10-relation transcription factors, Creb, Foxp1, and Ncor2, through the production of secondary messengers. While directly contributing to the production of IL-10, LANCL2 was also identified to contribute to the downregulation of Traf2 signaling and directly preventing inflammatory responses. Through use of a synergistic computational and experimental cycle, cellular and molecular mechanisms controlling regulatory responses were identified and validated. As a modifier of macrophage differentiation and regulatory pathways in general, LANCL2 may serve as a useful alternative to antibiotic eradication of *H*. *pylori*.

## Methods

### Ethics Statement

All experimental procedures were approved by the Institutional Animal Care and Use Committee (IACUC) of Virginia Tech and met or exceeded requirements of the Public Health Service/National Institutes of Health and the Animal Welfare Act. The IACUC approval IDs for the studies were 12-174-VBI and 15-147-VBI. C57BL/6J wild type and LANCL2-/-, and LANCL2^Myeloid^ mice were bred and maintained in experimental facilities at Virginia Polytechnic Institute and State University. Mice were housed two to five per cage on a ventilated rack in a room with a standard 12 hours on, 12 hours off light cycle. The animals were given ad libitum access to standard rodent chow and water. All mice were euthanized by carbon dioxide narcosis and a secondary cervical dislocation.

### *H*. *pylori* Infection Model

C57BL/6J wild-type, LANCL2-/-, LANCL2fl/fl; LysCre-, and LANCL2fl/fl; LysCre+ mice were challenged with 5x10^7^ colony forming units (CFU) of *H*. *pylori* strain SS1 in sterile 1X PBS via orogastric gavage. Mice were challenged in two doses, one occurring on day 0 of the study and the other on day 2. Non-challenged controls received an equal volume of sterile 1X PBS via gavage on each day [32]. Strain and dosing strategy were identical in both wild-type calibration data (n = 12) and LANCL2 validation data (n = 7) experiments.

### Sample Processing

Stomachs were collected at weeks 3, 11, and 20 post-infection for the assessment of bacterial loads and lamina propria immune cell composition. Stomach contents were removed, and samples were washed with sterile 1X PBS. Stomachs were divided into equal left and right proportions. The left half was used for immunophenotyping. Samples were washed in BD Cell Recovery Media to remove epithelial cells. The remaining tissue was digested in RPMI containing collagenase and DNase at 37°C while stirring. Samples were filtered and centrifuged. Remaining cells were re-suspended and purified in a Percoll gradient. Cells at the Percoll interface were collected and counted.

### Bacterial loads

The right half of the stomach was used for bacterial load quantification. Samples were homogenized in Brucella broth. The supernatant was serially diluted (1:10, 1:100, 1:1000) and plated on Columbia blood agar plates containing *Helicobacter* selective supplement. Plates were incubated in microaerophilic conditions using a Campygen gas generating pack for four days at 37°C. Colonies were counted and compared to sample weight for normalization.

### Real-time RT-PCR

mRNA was isolated from stomach sections using the RNeasy Mini Kit (Qiagen) as previously described [61]. qRT-PCR was run using primers designed for Ncor2 (forward: CTGCTGTCAACAACAGCTCTGATAC, reverse: GCTTCAGTGCCAGTGGGTTTAG), Foxp1 (forward: GCCAAGGCCTCCTAACAATTCA, reverse: TGGGCACTTGTCACTTCTTTCC) and Actb1 (forward: CCGAGGCATTGCTGACAGG, reverse: TGGAAGGTCGACAGTGAGGC) and ssoAdvanced SybrGreen (BioRad). Starting quantities of genes were calculated using standard curves and normalized to Actb1 starting quantity as previously described [62].

### Flow cytometry

Stomach lamina propria lymphocytes were plated in 96 well plates (6x10^5^ cells/well) and processed for immunophenotyping by flow cytometry as previously explained [63]. Briefly, cells were incubated with fluorochrome conjugated antibodies to extracellular markers: CD45, CD4, CD3, and CD25 for iTreg, CD45, CD4, CD3, CD25, and PD1 for Tr1 and CD45, CD11b, CD11c, CD64, F4/80, CX3CR1, and MHCII for macrophages. Samples needing a secondary staining were incubated with secondary antibodies, or streptavidin-conjugated fluorochrome. The samples were then fixed and permeabilized. Cells were incubated with antibodies to intracellular markers (IL10, FOXP3). Data was acquired with a BD LSRII flow cytometer and analyzed using FACS Diva software (BD Pharmingen).

### Computational modeling and SA

The model generation was a multi-step process, including the creation of a model network, calibration, and validation of the model equations, analysis of the model, and execution of *in silico* simulations. The structure of the computational model, which includes the species and their interactions, was constructed in CellDesigner, a Systems Biology Markup Language (SBML) compliant software. The first network was generated based on a combination of generated time course data and a thorough literature review and depicts the cellular host involving interactions between dendritic cells, T helper cells, macrophages, neutrophils, epithelial cells and commensal bacteria in response to *H*. *pylori* infection. The model was imported into Complex Pathway Simulator (COPASI) software [64]. In COPASI, the interactions and transitions were assigned ordinary differential equations representing multiple kinetics including mass action, simple activation and Hill-type activation and inhibition, available in S1 File. The resulting parameters in the tissue level model were estimated using Particle Swarm and Genetic algorithms with time course data generated through the mouse model at various time points post-infection utilizing methods as previously described [27,29]. The parameter search algorithms seek to minimize the sum of squares for the calibration dataset. To further train the model, a separate dataset containing results from additional time points post-infection was used as for validation. In the parameter estimation process, the sum of squares for the validation dataset is monitored but not minimized. Rather an increase in the sum of squares for the validation dataset is used as a stop criterion for the search algorithm which serves as a preventative measure against over-fitting. The intracellular macrophage model was steady state calibrated using methods as previously described [28,31]. Data was obtained from publications on the differentiation of macrophages and in vitro systems featuring the absence or over-activation of model nodes. Full calibration datasets for both models are available in S2 File. Parameters values are available in S1 File. Time course simulations were conducted using an LSODA deterministic method. Local sensitivities were calculated through numerical differentiation using a finite difference method with delta factor 0.001 and delta minimum 1x10^-12^. Global sensitivities were calculated based on sensitivity optimization method available from the high-performance computing based CONDOR-Copasi. *In silico* simulations of the effects of LANCL2 knockout were first generated within the second model to obtain an impairment ratio on the regulatory macrophages. The ratio was then used to modify parameters within the first model to observe changes in a time course simulation. Both tissue and intracellular level models will be deposited within the Biomodels.net database with identification numbers MODEL1611160001 and MODEL1611160002, respectively [65].

### Statistical analysis

A one way analysis of variance (ANOVA) was performed to determine significance in the data using a SAS (SAS Institute) general linear model procedure. Differences of p≤0.05 were considered significant. Data was comprised of multiple experiments. The number of samples for each group at each time point varied between five and eight. Data is displayed as mean values with error bars representing standard error of the mean and asterisks to mark significance.

## Supporting Information

S1 File Ordinary differential equations and parameter values. (DOCX)

S2 File Calibration database for estimation of model parameters. Database contains time course tissue level data and steady state macrophage data for the calibration of the two models.
(XLSX)

S3 File *In vivo* T cell responses to *H*. *pylori* infection. (PDF)

S4 File Characterization of LANCL2 knockout mice. (PDF)
