# Supplementary material for: Modeling the Role of Lanthionine Synthetase C-Like 2 (LANCL2) in the Modulation of Immune Responses to Helicobacter pylori Infection
Source: PLoS One. 2016 Dec 9;11(12):e0167440. doi: 10.1371/journal.pone.0167440 (PMC5147901; doi:10.1371/journal.pone.0167440)
Supplement: S1 File — (DOCX) [file pone.0167440.s001.docx]

**Equations for tissue-level model of *H. pylori* infection**

$$\frac{d[E]}{dt}=\left( \frac{p_{1}*\left[ E \right]}{\omega_{1}+\left[ E \right]} \right)-\left( \left[ E \right]*\left( k_{1}*\left[ Th1 \right]+k_{2}*\left[ HP_{Lumen} \right]-k_{3}\left[ IL10 \right] \right) \right)-(d_{1}*\left[ E \right])$$

$$\frac{d[Edamaged]}{dt}=\left( \left[ E \right]*\left( k_{1}*\left[ Th1 \right]+k_{2}*\left[ HP_{Lumen} \right]-k_{3}\left[ IL10 \right] \right) \right)-(d_{2}*\left[ Edead \right])$$

$$\frac{d[Edead]}{dt}=(d_{1}*\left[ E \right])$$

$$\frac{d[Edamageddead]}{dt}=(d_{2}*\left[ Edead \right])$$

$$\frac{d[iDC_{epithelium}]}{dt}=-\left( k_{4}*\left[ {HP}_{Lumen} \right]*\left[ {iDC}_{epithelium} \right] \right)-\left( \sigma_{1}*\left[ iDC_{epithelium} \right]-\sigma_{2}*\left[ iDC_{LP} \right] \right)-\left( k_{5}*\left[ {TolB}_{Lumen} \right]*\left[ {iDC}_{epithelium} \right] \right)-\left( k_{6}*\left[ {HP}_{Lumen} \right]*\left[ {iDC}_{epithelium} \right] \right)$$

$$\frac{d[{eDC}_{LP}]}{dt}= -\left( \sigma_{3}*\left[ {eDC}_{LP} \right] \right)+\left( k_{4}*\left[ {HP}_{Lumen} \right]*\left[ {iDC}_{epithelium} \right] \right)+\left( k_{7}*\left[ {HP}_{LP} \right]*\left[ {iDC}_{LP} \right] \right)- (d_{3}*(\left[ {iTreg}_{LP} \right]+1)*\left[ {eDC}_{LP} \right])$$

$$\frac{d[{tDC}_{LP}]}{dt}= (k_{5}*\left[ {TolB}_{Lumen} \right]*\left[ {iDC}_{epithelium} \right])- (d_{4}*\left[ {tDC}_{LP} \right])-\left( \sigma_{4}*\left[ {tDC}_{LP} \right] \right)+\left( k_{6}*\left[ {HP}_{Lumen} \right]*\left[ {iDC}_{epithelium} \right] \right)+(k_{8}*(\left[ {TolB}_{LP} \right]+1)*\left[ {iDC}_{LP} \right])+(k_{9}*(\left[ {HP}_{LP} \right]+1)*\left[ {iDC}_{LP} \right])$$

$$\frac{d[eDC_{gln}]}{dt}=(\sigma_{3}*\left[ eDC_{LP} \right]-d_{5}*\left[ eDC_{gln} \right])$$

$$\frac{d[tDC_{gln}]}{dt}=(\sigma_{4}*\left[ tDC_{LP} \right]-d_{6}*\left[ tDC_{gln} \right])$$

$$\frac{d[iDCdead]}{dt}=\left( d_{7}*\left[ iDC_{LP} \right]-k_{10}*\left[ iDCdead \right] \right)$$

$$\frac{d[eDCdead_{LP}]}{dt}=\left( \left[ eDC_{LP} \right]*\left( \left[ iTreg_{LP} \right]+1 \right)*d_{3} \right)$$

$$\frac{d[{tDCdead}_{LP}]}{dt}=\left( d_{4}*\left[ {tDC}_{LP} \right] \right)$$

$$\frac{d[eDC_{gln}dead]}{dt}=d_{5}*\left[ eDC_{gln} \right]$$

$$\frac{d[tDC_{gln}dead]}{dt}=d_{6}*\left[ tDC_{gln} \right]$$

$$\frac{d[{HP}_{Lumen}]}{dt}= -\left( \left[ {HP}_{Lumen} \right]*\left( \left[ Edamaged \right]+1 \right)*\sigma_{5} \right)+\left( \frac{p_{2}*\left[ {HP}_{Lumen} \right]}{\omega_{2}+ \left[ {HP}_{Lumen} \right]} \right)-\left( \left[ {HP}_{Lumen} \right]*d_{8}*\left[ Edamaged \right]+d_{9}*\left[ {IFN}_{\gamma} \right] \right)-(k_{4}*\left[ {HP}_{Lumen} \right]*\left[ {iDC}_{epithelium} \right])-(k_{6}*\left[ {HP}_{Lumen} \right]*\left[ {iDC}_{epithelium} \right])$$

$$\frac{d[{HP}_{LP}]}{dt}= \left( \left[ {HP}_{Lumen} \right]*\left( \left[ Edamaged \right]+1 \right)*\sigma_{5} \right)+\left( \frac{p_{3}*\left[ {HP}_{LP} \right]}{\omega_{3}+ \left[ {HP}_{LP} \right]} \right)-\left( \left[ {HP}_{LP} \right]*d_{10}*\left[ Edamaged \right]+d_{11}*\left[ {IFN}_{\gamma} \right] \right)-(k_{11}*\left[ {HP}_{LP} \right]*\left[ Monocytes \right])$$

$$\frac{d[{HPdead}_{Lumen}]}{dt}=\left( \left[ {HP}_{Lumen} \right]*d_{8}*\left[ Edamaged \right]+d_{9}*\left[ {IFN}_{\gamma} \right] \right)$$

$$\frac{d[{HPdead}_{LP}]}{dt}=\left( \left[ {HP}_{LP} \right]*d_{10}*\left[ Edamaged \right]+d_{11}*\left[ {IFN}_{\gamma} \right] \right)$$

$$\frac{d[IFN_{\gamma}]}{dt}=\left( \frac{\left[ pIFN_{\gamma} \right]*p_{4}*\left[ Th1_{LP} \right]}{\omega_{4}+\left[ IL10 \right]} \right)-(d_{12}*\left[ IFN_{\gamma} \right])$$

$$\frac{d[IL10]}{dt}= \left( \left[ pIL10 \right]*(k_{12}*\left[ M_{reg} \right]+k_{13}*[Tr1] \right)-\left( d_{13}*\left[ IL10 \right]) \right)$$

$$\frac{d[M_{reg}]}{dt}= \left( k_{14}*\left[ Monocytes \right]*\left[ TolB_{LP} \right] \right)+ (k_{11}*\left[ Monocytes \right]*\left[ {HP}_{LP} \right])-\left( \left[ M_{reg} \right]*(d_{14}*\left[ iTreg_{LP} \right]+d_{15}*\left[ M_{reg} \right]+d_{16}*\left[ Monocytes \right] \right)$$

$$\frac{d[Monocytes]}{dt}=-\left( k_{14}*\left[ Monocytes \right]*\left[ TolB_{LP} \right] \right)- \left( k_{11}*\left[ Monocytes \right]*\left[ {HP}_{LP} \right] \right)-\left( \left[ Monocytes \right]*d_{17}*\left[ Edamaged \right]+d_{18}*\left[ eDC_{LP} \right] \right)+(k_{15}+\frac{p_{5}*\left[ Edamaged \right]^{\alpha_{1}}}{\left[ Edamaged \right]^{\alpha_{1}}+{\omega_{5}}^{\alpha_{1}}})$$

$$\frac{d[M_{reg}dead]}{dt}=\left( \left[ M_{reg} \right]*(d_{14}*\left[ iTreg_{LP} \right]+d_{15}*\left[ M_{reg} \right]+d_{16}*\left[ Monocytes \right] \right)$$

$$\frac{d[Monocytesdead]}{dt}=\left( \left[ Monocytes \right]*d_{17}*\left[ Edamaged \right]+d_{18}*\left[ eDC_{LP} \right] \right)$$

$$\frac{d[{Th1}_{LP}]}{dt}= \left( \sigma_{6}*\left[ {Th1}_{GLN} \right] \right)-(\left( d_{19}*\left[ {Th1}_{LP} \right]) \right)$$

$$\frac{d[Th1_{gln}]}{dt}=(\left[ nT \right]*\left( \left[ eDC_{gln} \right]*k_{16}-\left[ IL10 \right]*k_{17} \right)-\sigma_{6}*\left[ Th1_{gln} \right]-d_{20}*[Th1_{gln}]$$

$$\frac{d[{Th1dead}_{LP}]}{dt}=\left( d_{19}*\left[ {Th1}_{LP} \right] \right)$$

$$\frac{d[{Th17}_{LP}]}{dt}= \left( \sigma_{7}*\left[ {Th17}_{GLN} \right] \right)-\left( d_{21}*\left[ {Th17}_{LP} \right])+(\left[ iTreg_{LP} \right]*(k_{18}*\left[ Th{17}_{LP} \right]+k_{19}*\left[ Th1_{LP} \right]+k_{20}*\left[ Edamaged \right]+k_{21}*[eDC_{LP}] \right))-(\left[ Th{17}_{LP} \right]*(k_{22}*\left[ iTreg_{LP} \right]+k_{23}*\left[ tDC_{LP} \right]))$$

$$\frac{d[{Th17}_{gln}]}{dt}=\left( \left[ nT \right]*(\left[ eDC_{gln} \right]+1)*k_{24} \right)-\sigma_{7}*\left[ {Th17}_{gln} \right]-d_{22}*\left[ {Th17}_{gln} \right]+(\left[ iTreg \right]*\left( k_{25}*\left[ Th1_{gln} \right]+k_{26}*\left[ eDC_{gln} \right]+k_{27}*\left[ Th{17}_{gln} \right] \right)-(\left[ Th17 \right]*(k_{28}*\left[ tDC_{gln} \right]+k_{29}*\left[ iTreg_{gln} \right])$$

$$\frac{d[{Th17dead}_{LP}]}{dt}=\left( d_{21}*\left[ {Th17}_{LP} \right] \right)$$

$$\frac{d[{iTreg}_{LP}]}{dt}= \left( \sigma_{8}*\left[ {iTreg}_{GLN} \right] \right)-\left( d_{23}*\left[ {iTreg}_{LP} \right])-\left( \left[ iTreg_{LP} \right]*(k_{18}*\left[ Th{17}_{LP} \right]+k_{19}*\left[ Th1_{LP} \right]+k_{20}*\left[ Edamaged \right]+k_{21}*[eDC_{LP}] \right))-(\left[ Th{17}_{LP} \right]*(k_{22}*\left[ iTreg_{LP} \right]+k_{23}*\left[ tDC_{LP} \right] \right))$$

$$\frac{d[iTreg_{gln}]}{dt}=\left( \left[ nT \right]*\left[ tDC_{gln} \right]*k_{30} \right)-\sigma_{8}*\left[ iTreg_{gln} \right]-d_{24}*\left[ iTreg_{gln} \right]-+(\left[ iTreg \right]*\left( k_{25}*\left[ Th1_{gln} \right]+k_{26}*\left[ eDC_{gln} \right]+k_{27}*\left[ Th{17}_{gln} \right] \right)-(\left[ Th17 \right]*(k_{28}*\left[ tDC_{gln} \right]+k_{29}*\left[ iTreg_{gln} \right])$$

$$\frac{d[{iTregdead}_{LP}]}{dt}=\left( d_{23}*\left[ {iTreg}_{LP} \right] \right)$$

$$\frac{d[Tr1]}{dt}=(\left[ M_{reg} \right]*k_{31}*\left( \left[ IL10 \right]*k_{32}+1 \right)-(d_{25}*\left[ Tr1 \right])$$

$$\frac{d[{TolB}_{Lumen}]}{dt}= -\left( \left[ {TolB}_{Lumen} \right]*\left( \left[ Edamaged \right]+1 \right)*\sigma_{9} \right)-(k_{5}*\left[ {TolB}_{Lumen} \right]*\left[ {iDC}_{epithelium} \right])$$

$$\frac{d[{TolB}_{LP}]}{dt}= \left( \left[ {TolB}_{Lumen} \right]*\left( \left[ Edamaged \right]+1 \right)*\sigma_{9} \right)-(k_{14}*\left[ {TolB}_{LP} \right]*\left[ Monocytes \right])$$

**Parameter descriptions and values for tissue-level model.** Symbol, value, units and associated function provided for each parameter within the model. Parameter symbols are grouped by purpose within an equation and given a unique number. d parameters are death and degradation rates, σ parameters are migration rates, p parameters are production rates, k parameters are general rate constants, α parameters are exponents, and ω parameters are specialized concentration constants.

| **Parameter** | **Value** | **Units** | **Function** |
| --- | --- | --- | --- |
| d1 | 0.05 | 1/s | E death |
| d2 | 0.049838 | 1/d | Edamaged death |
| d3 | 4.39E-07 | l/(#*d) | eDC LP death |
| d4 | 0.021112 | 1/d | tDC LP death |
| d5 | 0.005276 | 1/d | eDC GLN death |
| d6 | 0.009681 | 1/d | tDC GLN death |
| d7 | 0.1 | 1/d | iDC death |
| d8 | 8.11E-09 | l/(#*d) | HP lumen death |
| d9 | 5.55E-07 | l/(#*d) | HP lumen death |
| d10 | 1.25E-06 | l/(#*d) | HP LP death |
| d11 | 5.50E-06 | l/(#*d) | HP LP death |
| d12 | 0.11825 | 1/d | IFNg degradation |
| d13 | 0.1 | 1/d | IL10 degradation |
| d14 | 2.10E-08 | l/(#*d) | Mreg death |
| d15 | 2.10E-08 | l/(#*d) | Mreg death |
| d16 | 7.50E-09 | l/(#*d) | Mreg death |
| d17 | 7.46E-12 | l/(#*d) | Monocytes death |
| d18 | 7.46E-14 | l/(#*d) | Monocytes death |
| d19 | 53.8055 | 1/d | Th1 death |
| d20 | 0.167387 | 1/d | Th1 GLN death |
| d21 | 4.28394 | 1/d | Th17 death |
| d22 | 19.6785 | 1/d | Th17 GLN death |
| d23 | 0.003244 | 1/d | iTreg death |
| d24 | 0.037753 | 1/d | iTreg GLN death |
| d25 | 0.1 | 1/d | Tr1 death |

| **Parameter** | **Value** | **Units** | **Function** |
| --- | --- | --- | --- |
| k1 | 2.50E-09 | l/(#*d) | Epithelial damage |
| k2 | 2.50E-10 | l/(#*d) | Epithelial damage |
| k3 | 5.00E-08 | l/(#*d) | Epithelial damage |
| k4 | 5.81E-10 | l^2/(#*d) | eDC stimulation |
| k5 | 0.1 | l^2/(#*d) | TolB DC uptake |
| k6 | 7.86E-09 | l^2/(#*d) | tDC stimulation |
| k7 | 1.00E-08 | l/(#*d) | eDC stimulation LP HP |
| k8 | 0.1 | l/(#*d) | tDC stimulation LP TolB |
| k9 | 1.84E-08 | l/(#*d) | tDC stimulation LP HP |
| k10 | 0.1 | 1/d | iDC death |
| k11 | 1.27E-08 | l/(#*d) | HP monocytes uptake |
| k12 | 0.024 | l/(#*d) | IL10 production |
| k13 | 0.024 | l/(#*d) | IL10 production |
| k14 | 0.1 | l/(#*d) | TolB monocytes uptake |
| k15 | 350 | #/(l*d) | Monocytes production |
| k16 | 3.62E-05 | l/(#*d) | Th1 differentiation |
| k17 | 3.00E-05 | l/(#*d) | Th1 differentiation |
| k18 | 0.263435 | l/(#*d) | Treg to Th17 lp |
| k19 | 7.93E-05 | l/(#*d) | Treg to Th17 lp |
| k20 | 0.000185 | l/(#*d) | Treg to Th17 lp |
| k21 | 0.000743 | l/(#*d) | Treg to Th17 lp |
| k22 | 0.040876 | l/(#*d) | Th17 to Treg lp |
| k23 | 0.040876 | l/(#*d) | Th17 to Treg lp |
| k24 | 5.98E-06 | l/(#*d) | Th17 differentiation |
| k25 | 3.36E-06 | l/(#*d) | Treg to Th17 gln |
| k26 | 3.36E-06 | l/(#*d) | Treg to Th17 gln |
| k27 | 9.15E-08 | l/(#*d) | Treg to Th17 gln |
| k28 | 5.00E-08 | l/(#*d) | Th17 to Treg gln |
| k29 | 5.00E-08 | l/(#*d) | Th17 to Treg gln |
| k30 | 1.51E-06 | l/(#*d) | Treg differentiation |
| k31 | 0.047568 | l/d | Tr1 differentiation |
| k32 | 1.00E-06 | l/# | Tr1 differentiation |

| **Parameter** | **Value** | **Units** | **Function** |
| --- | --- | --- | --- |
| σ1 | 0.1 | l/d | iDC migration |
| σ2 | 0.1 | l/d | iDC migration |
| σ3 | 0.092119 | l/d | eDC migration |
| σ4 | 0.01 | l/d | tDC migration |
| σ5 | 1.74E-07 | l/(#*d) | HP migration |
| σ6 | 12.7624 | l/d | Th1 migration |
| σ7 | 0.006755 | l/d | Th17 migration |
| σ8 | 0.280925 | l/d | iTreg migration |
| σ9 | 0.1 | l/(#*d) | TolB migration |
| p1 | 40000.1 | l/(#*d) | E production |
| p2 | 100550 | l/(#*d) | HP lumen production |
| p3 | 58731.9 | l/(#*d) | HP LP production |
| p4 | 5.07025 | 1/d | IFNg production |
| p5 | 68855.3 | 1 | Monocytes production |
| α1 | 1.1 | 1 | Monocytes production |
| ω1 | 100 | #/l | E production |
| ω2 | 100 | #/l | HP lumen production |
| ω3 | 100.55 | #/l | HP LP production |
| ω4 | 1000 | #/l | IFNg production |
| ω5 | 440600 | #/l | Monocytes production |

**Equations for intracellular macrophage model**

$$\frac{d[Akt]}{dt}=V_{f1}*[pAkt]\left( \frac{{[LANCL2]}^{\alpha_{1}}}{{[LANCL2]}^{\alpha_{1}}+{k_{1}}^{\alpha_{1}}}+\frac{{[CX3CR1]}^{\alpha_{2}}}{{[CX3CR1]}^{\alpha_{2}}+{k_{2}}^{\alpha_{2}}}+\frac{{[DAP12]}^{\alpha_{3}}}{{[DAP12]}^{\alpha_{3}}+{k_{3}}^{\alpha_{3}}} \right)-V_{r1}*[Akt]$$

$$\frac{d[pAkt]}{dt}={-V}_{f1}*[pAkt]\left( \frac{{[LANCL2]}^{\alpha_{1}}}{{[LANCL2]}^{\alpha_{1}}+{k_{1}}^{\alpha_{1}}}+\frac{{[CX3CR1]}^{\alpha_{2}}}{{[CX3CR1]}^{\alpha_{2}}+{k_{2}}^{\alpha_{2}}}+\frac{{[DAP12]}^{\alpha_{3}}}{{[DAP12]}^{\alpha_{3}}+{k_{3}}^{\alpha_{3}}} \right)-V_{r1}*[Akt]$$

$$\frac{d[eCa]}{dt}=-V_{f2}*\left[ ERK \right]*\left[ eCa \right]+V_{r2}*[iCa]$$

$$\frac{d[iCa]}{dt}=V_{f2}*\left[ ERK \right]*\left[ eCa \right]-V_{r2}*[iCa]$$

$$\frac{d[cAMP]}{dt}=V_{f3}*\left[ AC \right]*\left( 1+\frac{{[LANCL2]}^{\alpha_{4}}}{{[LANCL2]}^{\alpha_{4}}+{k_{4}}^{\alpha_{4}}} \right)-d_{1}*[cAMP]$$

$$\frac{d[CREB]}{dt}=V_{f4}*[pCREB]\left( \frac{{[PKA]}^{\alpha_{5}}}{{[PKA]}^{\alpha_{5}}+{k_{5}}^{\alpha_{5}}}+\frac{{[iCa]}^{\alpha_{6}}}{{[iCa]}^{\alpha_{6}}+{k_{6}}^{\alpha_{6}}} \right)-V_{r4}*[CREB]$$

$$\frac{d[pCREB]}{dt}={-V}_{f4}*\left[ pCREB \right]\left( \frac{\left[ PKA \right]^{\alpha_{5}}}{\left[ PKA \right]^{\alpha_{5}}+{k_{5}}^{\alpha_{5}}}+\frac{\left[ iCa \right]^{\alpha_{6}}}{\left[ iCa \right]^{\alpha_{6}}+{k_{6}}^{\alpha_{6}}} \right)+V_{r4}*[CREB]$$

$$\frac{d[CSF1R]}{dt}=V_{f5}*[pCSF1R]\left( \frac{\left[ FOXP1 \right]^{\alpha_{7}}}{\left[ FOXP1 \right]^{\alpha_{7}}+{k_{7}}^{\alpha_{7}}}+\frac{\left[ M-CSF \right]^{\alpha_{8}}}{\left[ M-CSF \right]^{\alpha_{8}}+{k_{8}}^{\alpha_{8}}} \right)-V_{r5}*[CSF1R]$$

$$\frac{d[pCSF1R]}{dt}=-V_{f5}*\left[ pCSF1R \right]\left( \frac{\left[ FOXP1 \right]^{\alpha_{7}}}{\left[ FOXP1 \right]^{\alpha_{7}}+{k_{7}}^{\alpha_{7}}}+\frac{\left[ M-CSF \right]^{\alpha_{8}}}{\left[ M-CSF \right]^{\alpha_{8}}+{k_{8}}^{\alpha_{8}}} \right)+V_{r5}*[CSF1R]$$

$$\frac{d[CX3CL1]}{dt}=V_{f6}*\left[ pCX3CL1 \right]*\left( \beta_{1}+\frac{\left[ KLF4 \right]^{\alpha_{9}}}{\left[ KLF4 \right]^{\alpha_{9}}+{k_{9}}^{\alpha_{9}}} \right)-d_{2}*[CX3CL1]$$

$$\frac{d[CX3CR1]}{dt}=V_{f7}*\left( \frac{\left[ NFAT \right]^{\alpha_{10}}}{\left[ NFAT \right]^{\alpha_{10}}+{k_{10}}^{\alpha_{10}}}+\frac{\left[ CX3CL1 \right]^{\alpha_{11}}}{\left[ CX3CL1 \right]^{\alpha_{11}}+{k_{11}}^{\alpha_{11}}} \right)-V_{r7}*[CX3CR1]$$

$$\frac{d[pCX3CR1]}{dt}=-V_{f7}*\left( \frac{\left[ NFAT \right]^{\alpha_{10}}}{\left[ NFAT \right]^{\alpha_{10}}+{k_{10}}^{\alpha_{10}}}+\frac{\left[ CX3CL1 \right]^{\alpha_{11}}}{\left[ CX3CL1 \right]^{\alpha_{11}}+{k_{11}}^{\alpha_{11}}} \right)+V_{r7}*[CX3CR1]$$

$$\frac{d[DAP12]}{dt}=\mu_{1}*\left[ SIRPb1 \right]-d_{3}*\left[ DAP12 \right]$$

$$\frac{d[ERK]}{dt}=V_{f8}*\left( \frac{{[CX3CR1]}^{\alpha_{12}}}{{[CX3CR1]}^{\alpha_{12}}+{k_{12}}^{\alpha_{12}}}+\frac{{[DAP12]}^{\alpha_{13}}}{{[DAP12]}^{\alpha_{13}}+{k_{13}}^{\alpha_{13}}} \right)-V_{r8}*[ERK]$$

$$\frac{d[pERK]}{dt}=-V_{f8}*\left( \frac{\left[ CX3CR1 \right]^{\alpha_{12}}}{\left[ CX3CR1 \right]^{\alpha_{12}}+{k_{12}}^{\alpha_{12}}}+\frac{\left[ DAP12 \right]^{\alpha_{13}}}{\left[ DAP12 \right]^{\alpha_{13}}+{k_{13}}^{\alpha_{13}}} \right)+V_{r8}*[ERK]$$

$$\frac{d\left[ Fbxo7 \right]}{dt}=V_{f9}*\left[ pFbxo7 \right]*\left( \beta_{2}+\frac{\left[ LANCL2 \right]^{\alpha_{14}}}{\left[ LANCL2 \right]^{\alpha_{14}}+{k_{14}}^{\alpha_{14}}} \right)-V_{r9}*\left[ Fbxo7 \right]$$

$$\frac{d[pFbxo7]}{dt}=-V_{f9}*\left[ pFbxo7 \right]*\left( \beta_{2}+\frac{\left[ LANCL2 \right]^{\alpha_{14}}}{\left[ LANCL2 \right]^{\alpha_{14}}+{k_{14}}^{\alpha_{14}}} \right)+V_{r9}*\left[ Fbxo7 \right]$$

$$\frac{d[FOXP1]}{dt}=V_{f10}*\left[ pFOXP1 \right]*\left( \frac{\left[ NCOR2 \right]^{\alpha_{15}}}{\left[ NCOR2 \right]^{\alpha_{15}}+{k_{15}}^{\alpha_{15}}} \right)-V_{r10}*[FOXP1]$$

$$\frac{d[pFOXP1]}{dt}=-V_{f10}*\left[ pFOXP1 \right]*\left( \frac{\left[ NCOR2 \right]^{\alpha_{15}}}{\left[ NCOR2 \right]^{\alpha_{15}}+{k_{15}}^{\alpha_{15}}} \right)+V_{r10}*[FOXP1]$$

$$\frac{d[IL1B]}{dt}=V_{f11}*\left[ pIL1B \right]*\left( \frac{{k_{16}}^{\alpha_{16}}}{{k_{16}}^{\alpha_{16}}+\left[ IL10R \right]^{\alpha_{16}}}+\frac{{k_{17}}^{\alpha_{17}}}{{k_{17}}^{\alpha_{17}}+\left[ M_{reg} \right]^{\alpha_{17}}} \right)*\left( \frac{\left[ NFkB \right]^{\alpha_{18}}}{\left[ NFkB \right]^{\alpha_{18}}+{k_{18}}^{\alpha_{18}}}+\frac{\left[ NFAT \right]^{\alpha_{19}}}{\left[ NFAT \right]^{\alpha_{19}}+{k_{19}}^{\alpha_{19}}} \right)-d_{4}*[IL1B]$$

$$\frac{d[IL10]}{dt}=V_{f12}*\left[ pIL10 \right]*\frac{{k_{20}}^{\alpha_{20}}}{{k_{20}}^{\alpha_{20}}+\left[ IL1B \right]^{\alpha_{20}}}*\left( \frac{\left[ CREB \right]^{\alpha_{21}}}{\left[ CREB \right]^{\alpha_{21}}+{k_{21}}^{\alpha_{21}}}+\frac{\left[ FOXP1 \right]^{\alpha_{22}}}{\left[ FOXP1 \right]^{\alpha_{22}}+{k_{22}}^{\alpha_{22}}} +\frac{\left[ M_{reg} \right]^{\alpha_{23}}}{\left[ M_{reg} \right]^{\alpha_{23}}+{k_{23}}^{\alpha_{23}}} \right)-d_{5}*[IL10]$$

$$\frac{d[IL10R]}{dt}=V_{f13}*\left( \beta_{3}+\frac{\left[ IL10 \right]^{\alpha_{24}}}{\left[ IL10 \right]^{\alpha_{24}}+{k_{24}}^{\alpha_{24}}} \right)-V_{r13}*[IL10R]$$

$$\frac{d[pIL10R]}{dt}=-V_{f13}*\left( \beta_{3}+\frac{\left[ IL10 \right]^{\alpha_{24}}}{\left[ IL10 \right]^{\alpha_{24}}+{k_{24}}^{\alpha_{24}}} \right)+V_{r13}*[IL10R]$$

$$\frac{d\left[ KLF4 \right]}{dt}=V_{f14}*\left[ pKLF4 \right]*\left( \beta_{4}+\frac{\left[ PKA \right]^{\alpha_{25}}}{\left[ PKA \right]^{\alpha_{25}}+{k_{25}}^{\alpha_{25}}} \right)-V_{r14}*\left[ KLF4 \right]$$

$$\frac{d[pKLF4]}{dt}={-V}_{f14}*\left[ pKLF4 \right]*\left( \beta_{4}+\frac{\left[ PKA \right]^{\alpha_{25}}}{\left[ PKA \right]^{\alpha_{25}}+{k_{25}}^{\alpha_{25}}} \right)+V_{r14}*\left[ KLF4 \right]$$

$$\frac{d[LANCL2]}{dt}=V_{f15}*[pLANCL2]\left( \beta_{5}+\frac{\left[ ABA \right]^{\alpha_{26}}}{\left[ ABA \right]^{\alpha_{26}}+{k_{26}}^{\alpha_{26}}} \right)-V_{r15}*[LANCL2]$$

$$\frac{d[pLANCL2]}{dt}=-V_{f15}*[pLANCL2]\left( \beta_{5}+\frac{\left[ ABA \right]^{\alpha_{26}}}{\left[ ABA \right]^{\alpha_{26}}+{k_{26}}^{\alpha_{26}}} \right)-V_{r15}*[LANCL2]$$

$$\frac{d[M-CSF]}{dt}=V_{f16}*\left[ pM-CSF \right]*\left( \beta_{6}+\frac{\left[ NFAT \right]^{\alpha_{27}}}{\left[ NFAT \right]^{\alpha_{27}}+{k_{27}}^{\alpha_{27}}} \right)-d_{6}*[M-CSF]$$

$$\frac{d\left[ M_{reg} \right]}{dt}=\frac{\mu_{2}*{[CX3CR1]}^{\alpha_{28}}*{[IL10R]}^{\alpha_{29}}*{[CSF1R]}^{\alpha_{30}}}{\left[ CX3CR1 \right]+\left[ IL10R \right]+\left[ CSF1R \right]+\omega_{1}}-d_{7}*[M_{reg}]$$

$$\frac{d\left[ Mono \right]}{dt}=-\frac{\mu_{2}*\left[ CX3CR1 \right]^{\alpha_{28}}*\left[ IL10R \right]^{\alpha_{29}}*\left[ CSF1R \right]^{\alpha_{30}}}{\left[ CX3CR1 \right]+\left[ IL10R \right]+\left[ CSF1R \right]+\omega_{1}}+d_{7}*[M_{reg}]$$

$$\frac{d[NFAT]}{dt}=V_{f17}*\left[ pNFAT \right]*\frac{{k_{31}}^{\alpha_{31}}}{{k_{31}}^{\alpha_{31}}+\left[ Akt \right]^{\alpha_{31}}}*\left( \beta_{7}+\frac{{[DAP12]}^{\alpha_{32}}}{{[DAP12]}^{\alpha_{32}}+{k_{32}}^{\alpha_{32}}} \right)-V_{r17}*[NFAT]$$

$$\frac{d[pNFAT]}{dt}=-V_{f17}*\left[ pNFAT \right]*\frac{{k_{31}}^{\alpha_{31}}}{{k_{31}}^{\alpha_{31}}+\left[ Akt \right]^{\alpha_{31}}}*\left( \beta_{7}+\frac{\left[ DAP12 \right]^{\alpha_{32}}}{\left[ DAP12 \right]^{\alpha_{32}}+{k_{32}}^{\alpha_{32}}} \right)+V_{r17}*[NFAT]$$

$$\frac{d[NFkB]}{dt}=V_{f18}*\left[ pNFkB \right]*\frac{{k_{33}}^{\alpha_{33}}}{{k_{33}}^{\alpha_{33}}+\left[ NCOR2 \right]^{\alpha_{33}}}\left( \beta_{8}+\frac{{[TRAF2]}^{\alpha_{34}}}{{[TRAF2]}^{\alpha_{34}}+{k_{34}}^{\alpha_{34}}} \right)-V_{r18}*[NFkB]$$

$$\frac{d[pNFkB]}{dt}={-V}_{f18}*\left[ pNFkB \right]*\frac{{k_{33}}^{\alpha_{33}}}{{k_{33}}^{\alpha_{33}}+\left[ NCOR2 \right]^{\alpha_{33}}}\left( \beta_{8}+\frac{\left[ TRAF2 \right]^{\alpha_{34}}}{\left[ TRAF2 \right]^{\alpha_{34}}+{k_{34}}^{\alpha_{34}}} \right)+V_{r18}*[NFkB]$$

$$\frac{d[NCOR2]}{dt}=V_{f19}*\left[ pNCOR2 \right]*\left( \beta_{9}+\frac{\left[ LANCL2 \right]^{\alpha_{35}}}{\left[ LANCL2 \right]^{\alpha_{35}}+{k_{35}}^{\alpha_{35}}} \right)-V_{r19}*[NCOR2]$$

$$\frac{d[pNCOR2]}{dt}=-V_{f19}*\left[ pNCOR2 \right]*\left( \beta_{9}+\frac{\left[ LANCL2 \right]^{\alpha_{35}}}{\left[ LANCL2 \right]^{\alpha_{35}}+{k_{35}}^{\alpha_{35}}} \right)+V_{r19}*[NCOR2]$$

$$\frac{d[PKA]}{dt}=V_{f20}*\left[ pPKA \right]*\left( \beta_{10}+\frac{{[cAMP]}^{\alpha_{36}}}{{[cAMP]}^{\alpha_{36}}+{k_{36}}^{\alpha_{36}}} \right)-V_{r20}*[PKA]$$

$$\frac{d[pPKA]}{dt}={-V}_{f20}*\left[ pPKA \right]*\left( \beta_{10}+\frac{\left[ cAMP \right]^{\alpha_{36}}}{\left[ cAMP \right]^{\alpha_{36}}+{k_{36}}^{\alpha_{36}}} \right)+V_{r20}*[PKA]$$

$$\frac{d[TRAF2]}{dt}=V_{f21}*\left[ pTRAF2 \right]*\frac{{k_{37}}^{\alpha_{37}}}{{k_{37}}^{\alpha_{37}}+\left[ Fbxo7 \right]^{\alpha_{37}}}*\left( \frac{\left[ IL1B \right]^{\alpha_{38}}}{\left[ IL1B \right]^{\alpha_{38}}+{k_{37}}^{\alpha_{38}}}+\frac{\left[ HP \right]^{\alpha_{39}}}{\left[ HP \right]^{\alpha_{39}}+{k_{39}}^{\alpha_{39}}} \right)-V_{r21}*[TRAF2]$$

$$\frac{d[pTRAF2]}{dt}={-V}_{f21}*\left[ pTRAF2 \right]*\frac{{k_{37}}^{\alpha_{37}}}{{k_{37}}^{\alpha_{37}}+\left[ Fbxo7 \right]^{\alpha_{37}}}*\left( \frac{\left[ IL1B \right]^{\alpha_{38}}}{\left[ IL1B \right]^{\alpha_{38}}+{k_{37}}^{\alpha_{38}}}+\frac{\left[ HP \right]^{\alpha_{39}}}{\left[ HP \right]^{\alpha_{39}}+{k_{39}}^{\alpha_{39}}} \right)+V_{r21}*[TRAF2]$$

**Parameter descriptions and values for intracellular macrophage model.** Symbol, value, units and associated function provided for each parameter within the model. Parameter symbols are grouped by purpose within an equation and given a unique number. d parameters are degradation rates, β parameters are unitless constants, μ parameters are specialized forward rates, ω parameters are specialized concentration constants, α parameters are exponents, Vf parameters are forward velocities, Vr parameters are reverse velocities, and k parameters are concentration constants.

| **Parameter** | **Value** | **Units** | **Function** |
| --- | --- | --- | --- |
| d1 | 0.1 | 1/s | cAMP degradation |
| d2 | 0.1 | 1/s | CX3CL1 degradation |
| d3 | 0.1 | 1/s | DAP12 degradation |
| d4 | 0.1 | 1/s | IL1B degradation |
| d5 | 0.1 | 1/s | IL10 degradation |
| d6 | 0.1 | 1/s | M-CSF degradation |
| d7 | 0.1589 | 1/s | Mreg differentiation |
| β1 | 0.1 | 1 | CX3CL1 production |
| β2 | 0.1 | 1 | Fbxo7 activation |
| β3 | 0 | 1 | IL10R activation |
| β4 | 0.1 | 1 | KLF4 activation |
| β5 | 0.1 | 1 | LANCL2 activation |
| β6 | 0.1 | 1 | M-CSF production |
| β7 | 0.1 | 1 | NFAT activation |
| β8 | 0.1 | 1 | NFkB activation |
| β9 | 0.1 | 1 | NCOR2 activation |
| β10 | 0.1 | 1 | PKA activation |
| μ1 | 0.038436 | 1/s | DAP12 activation |
| μ2 | 1.49275 | (l^2.43)/(s*mol^2.43) | Mreg differentiation |
| ω1 | 0.08075 | mol/l | Mreg differentiation |

| **Parameter** | **Value** | **Units** | **Function** |
| --- | --- | --- | --- |
| α1 | 1.136 | 1 | Akt activation |
| α2 | 1.136 | 1 | Akt activation |
| α3 | 1.136 | 1 | Akt activation |
| α4 | 1.0895 | 1 | cAMP production |
| α5 | 1.508 | 1 | CREB activation |
| α6 | 1.508 | 1 | CREB activation |
| α7 | 1.907 | 1 | CSF1R activation |
| α8 | 1.907 | 1 | CSF1R activation |
| α9 | 1 | 1 | CX3CL1 production |
| α10 | 1.6325 | 1 | CX3CR1 activation |
| α11 | 1.6325 | 1 | CX3CR1 activation |
| α12 | 1.0115 | 1 | ERK activation |
| α13 | 1.0115 | 1 | ERK activation |
| α14 | 1.349 | 1 | Fbxo7 activation |
| α15 | 1.193 | 1 | FOXP1 activation |
| α16 | 1.151 | 1 | IL1B production |
| α17 | 1.151 | 1 | IL1B production |
| α18 | 1.151 | 1 | IL1B production |
| α19 | 1.151 | 1 | IL1B production |
| α20 | 1.193 | 1 | IL10 production |
| α21 | 1.193 | 1 | IL10 production |
| α22 | 1.193 | 1 | IL10 production |
| α23 | 1.193 | 1 | IL10 production |
| α24 | 1.042 | 1 | IL10R activation |
| α25 | 1.1 | 1 | KLF4 activation |
| α26 | 1.643 | 1 | LANCL2 activation |
| α27 | 1.049 | 1 | M-CSF production |
| α28 | 1.001 | 1 | Mreg differentiation |
| α29 | 2.428 | 1 | Mreg differentiation |
| α30 | 1.001 | 1 | Mreg differentiation |
| α31 | 1.5755 | 1 | NFAT activation |
| α32 | 1.5755 | 1 | NFAT activation |
| α33 | 1.73699 | 1 | NFkB activation |
| α34 | 1.73699 | 1 | NFkB activation |
| α35 | 1.8185 | 1 | NCOR2 activation |
| α36 | 1.301 | 1 | PKA activation |
| α37 | 1.493 | 1 | TRAF2 activation |
| α38 | 1.493 | 1 | TRAF2 activation |
| α39 | 1.493 | 1 | TRAF2 activation |

| **Parameter** | **Value** | **Units** | **Function** |
| --- | --- | --- | --- |
| Vf1 | 0.1115 | 1/s | Akt activation |
| Vf2 | 0.3076 | l/(mol*s) | Calcium transport |
| Vf3 | 0.029 | 1/s | cAMP production |
| Vf4 | 0.1493 | 1/s | CREB activation |
| Vf5 | 0.185 | 1/s | CSF1R activation |
| Vf6 | 0.126 | 1/s | CX3CL1 production |
| Vf7 | 0.13385 | mol/(l*s) | CX3CR1 activation |
| Vf8 | 0.048701 | mol/(l*s) | ERK activation |
| Vf9 | 0.1235 | 1/s | Fbxo7 activation |
| Vf10 | 0.10955 | 1/s | FOXP1 activation |
| Vf11 | 0.467575 | 1/s | IL1B production |
| Vf12 | 1.66808 | 1/s | IL10 production |
| Vf13 | 0.169469 | 1/s | IL10R activation |
| Vf14 | 0.1 | 1/s | KLF4 activation |
| Vf15 | 0.1328 | 1/s | LANCL2 activation |
| Vf16 | 0.03888 | 1/s | M-CSF production |
| Vf17 | 0.11105 | 1/s | NFAT activation |
| Vf18 | 0.139789 | 1/s | NFkB activation |
| Vf19 | 0.124465 | 1/s | NCOR2 activation |
| Vf20 | 0.1199 | 1/s | PKA activation |
| Vf21 | 0.984949 | 1/s | TRAF2 activation |
| Vr1 | 0.1922 | 1/s | Akt activation |
| Vr2 | 0.1292 | 1/s | Calcium transport |
| Vr4 | 0.19685 | 1/s | CREB activation |
| Vr5 | 0.2 | 1/s | CSF1R activation |
| Vr7 | 0.1715 | 1/s | CX3CR1 activation |
| Vr8 | 0.1607 | 1/s | ERK activation |
| Vr9 | 0.19115 | 1/s | Fbxo7 activation |
| Vr10 | 0.31685 | 1/s | FOXP1 activation |
| Vr13 | 0.188 | 1/s | IL10R activation |
| Vr14 | 0.114 | 1/s | KLF4 activation |
| Vr15 | 0.1835 | 1/s | LANCL2 activation |
| Vr17 | 0.18755 | 1/s | NFAT activation |
| Vr18 | 0.481511 | 1/s | NFkB activation |
| Vr19 | 0.59075 | 1/s | NCOR2 activation |
| Vr20 | 0.18635 | 1/s | PKA activation |
| Vr21 | 0.262 | 1/s | TRAF2 activation |

| **Parameter** | **Value** | **Units** | **Function** |
| --- | --- | --- | --- |
| k1 | 0.01779 | mol/l | Akt activation |
| k2 | 0.11105 | mol/l | Akt activation |
| k3 | 0.1328 | mol/l | Akt activation |
| k4 | 0.185 | mol/l | cAMP production |
| k5 | 0.0635 | mol/l | CREB activation |
| k6 | 0.0728 | mol/l | CREB activation |
| k7 | 0.36647 | mol/l | CSF1R activation |
| k8 | 0.0551 | mol/l | CSF1R activation |
| k9 | 0.0678 | mol/l | CX3CL1 production |
| k10 | 0.19115 | mol/l | CX3CR1 activation |
| k11 | 0.05 | mol/l | CX3CR1 activation |
| k12 | 0.1043 | mol/l | ERK activation |
| k13 | 0.0851 | mol/l | ERK activation |
| k14 | 0.1536 | mol/l | Fbxo7 activation |
| k15 | 0.0836 | mol/l | FOXP1 activation |
| k16 | 0.06035 | mol/l | IL1B production |
| k17 | 0.0692 | mol/l | IL1B production |
| k18 | 0.06755 | mol/l | IL1B production |
| k19 | 0.0785 | mol/l | IL1B production |
| k20 | 0.0536 | mol/l | IL10 production |
| k21 | 0.0593 | mol/l | IL10 production |
| k22 | 0.09815 | mol/l | IL10 production |
| k23 | 0.01589 | mol/l | IL10 production |
| k24 | 0.463615 | mol/l | IL10R activation |
| k25 | 0.0743 | mol/l | KLF4 activation |
| k26 | 0.06395 | mol/l | LANCL2 activation |
| k27 | 0.13325 | mol/l | M-CSF production |
| k31 | 0.1307 | mol/l | NFAT activation |
| k32 | 0.1586 | mol/l | NFAT activation |
| k33 | 0.316437 | mol/l | NFkB activation |
| k34 | 0.015346 | mol/l | NFkB activation |
| k35 | 0.05315 | mol/l | NCOR2 activation |
| k36 | 0.0515 | mol/l | PKA activation |
| k37 | 0.09545 | mol/l | TRAF2 activation |
| k38 | 0.06815 | mol/l | TRAF2 activation |
| k39 | 0.01646 | mol/l | TRAF2 activation |
