# Supplementary material for: Modeling the Role of Lanthionine Synthetase C-Like 2 (LANCL2) in the Modulation of Immune Responses to Helicobacter pylori Infection
Source: PLoS One. 2016 Dec 9;11(12):e0167440. doi: 10.1371/journal.pone.0167440 (PMC5147901; doi:10.1371/journal.pone.0167440)
Supplement: S3 File — (PDF) [file pone.0167440.s003.pdf]

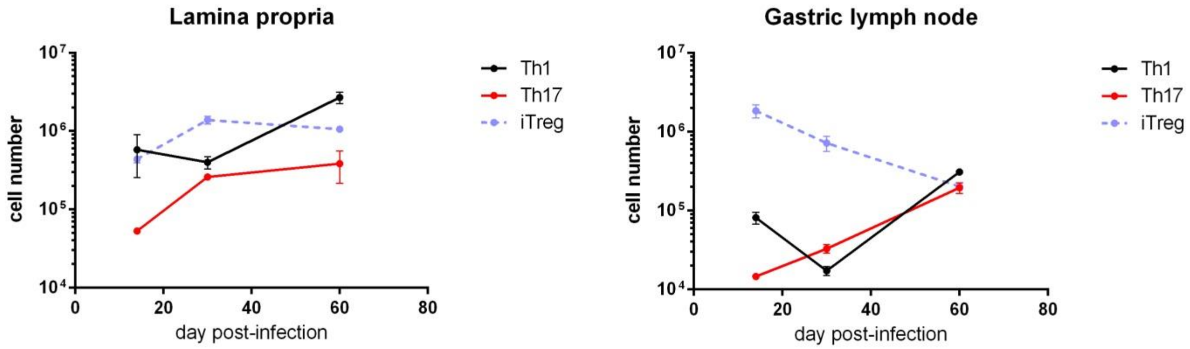

Fig. S3. *In vivo* T cell responses to *H. pylori* infection. Cell numbers of Th1 (CD4+CD3+Tbet+IFNγ+), Th17 (CD4+CD3+RORγT+IL17+) and iTreg (CD4+CD3+FOXP3+IL10+) in stomach lamina propria and gastric lymph node through time course of *H. pylori* (strain: SS1) infection in wild type mice.
