# Supplementary material for: Modeling the Role of Lanthionine Synthetase C-Like 2 (LANCL2) in the Modulation of Immune Responses to Helicobacter pylori Infection
Source: PLoS One. 2016 Dec 9;11(12):e0167440. doi: 10.1371/journal.pone.0167440 (PMC5147901; doi:10.1371/journal.pone.0167440)
Supplement: S4 File — (PDF) [file pone.0167440.s004.pdf]

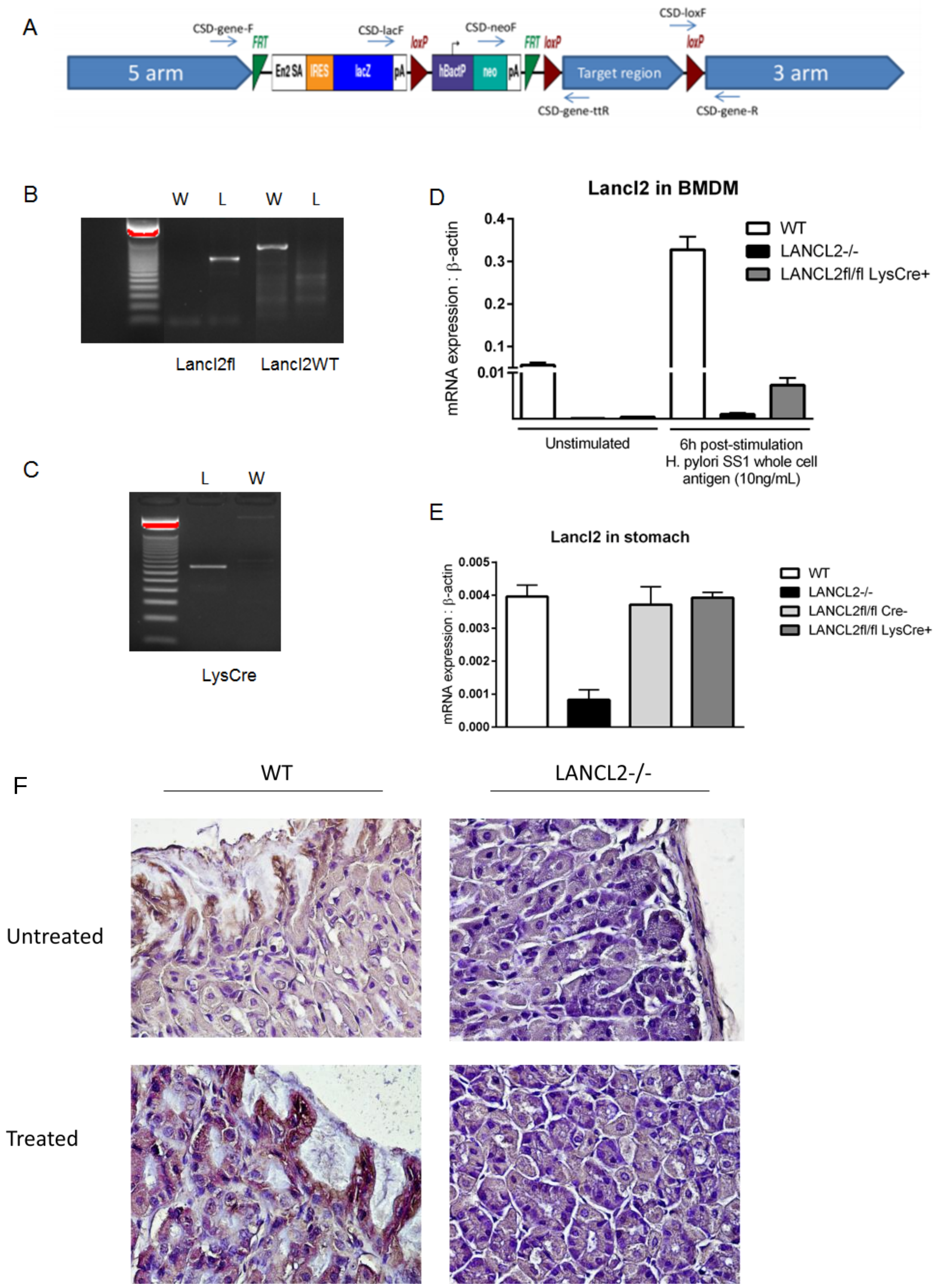

Figure S4. Characterization of LANCL2 knockout mice. Graphical scheme of genetic modification for knockout of LANCL2 (A). Representative images of PCR gels for floxed LANCL2 (B) and lysozyme cre (C) sites within Lancl2fl/fl LysCre+, L, and wild type, W, mice. Expression of LANCL2 in bone marrow derived macrophages in unstimulated and six hours post stimulation with H. pylori SS1 whole cell isolate (10ng/mL) in wild type, LANCL2-/-, and LANCL2fl/fl LysCre+ cells (D). Expression of LANCL2 in whole stomach RNA in WT, LANCL2-/-, LANCL2fl/flCre- and LANCL2fl/fl LysCre+ (E). Immunohistochemistry of wild-type and LANCL2-/- stomach with anti-LANCL2 antibody (MyBioSource, MBS154355) following five days of no treatment or treatment with LANCL2 ligand NSC61610 (F).
